# Combination treatment with telitacicept, cyclophosphamide and glucocorticoids for severe Granulomatous polyangiitis: a case report and literature review

**DOI:** 10.3389/fimmu.2023.1298650

**Published:** 2023-12-01

**Authors:** Liqi Huang, Wenjian Lin, Yu Liu, Junfeng Zhu, Yun Li, Zhihua Zheng, Chun Tang

**Affiliations:** ^1^ Department of Nephrology, Center of Nephrology and Urology, The Seventh Affiliated Hospital, Sun Yat-sen University, Shenzhen, China; ^2^ Department of Pathology, The Seventh Affiliated Hospital of Sun Yat-Sen University, Shenzhen, Guangdong, China; ^3^ Department of Thoracic Surgery, The Seventh Affiliated Hospital of Sun Yat-Sen University, Shenzhen, Guangdong, China

**Keywords:** granulomatous polyangiitis, rapidly progressive glomerulonephritis, telitacicept, case report, combination treatment

## Abstract

Granulomatous polyangiitis (GPA) is a rare autoimmune disease that can involve multiple systems throughout the body, including the ear, nose, upper and lower respiratory tracts. It is classified as an antineutrophil cytoplasmic antibody (ANCA)-associated vasculitis. Telitacicept is a novel recombinant fusion protein targeting B-lymphocyte stimulator (BLyS). Telitacicept can inhibit the development and maturation of abnormal B cells by blocking BLyS, and inhibit the production of antibodies by abnormal plasma cells by blocking APRIL (A proliferation-inducing ligand), which is expected to become a new drug for the treatment of GPA. We report a 64-year-old man diagnosed at our hospital with GPA involving multiple systems including kidneys, lungs, nose and ears. Renal involvement was severe, with a clinical characteristic of rapidly progressive glomerulonephritis and a pathologic manifestation of crescentic nephritis with plasma cell infiltration. The patient was treated with hormones, immunoglobulins and cyclophosphamide (CYC) with the addition of telitacicept and a rapid reduction in hormone dosage. The patient’s renal function improved significantly within a short period of time, and his hearing and lung lesions improved significantly. At the same time, he did not develop serious infections and other related complications. Our report suggests that short-term control of the patient’s conditions is necessary in GPA patients with organ-threatening disease. Telitacicept combined with CYC and glucocorticoids may be an induction therapy with safety and feasibility. However, more clinical trials are needed to validate the efficacy and safety of the therapeutic regimen.

## 1 Introduction

Granulomatous polyangiitis (GPA) is characterized by necrotizing granulomatous polyangiitis involving the ear, nose, upper and lower airways. Necrotizing vasculitis primarily involves small and medium-sized vessels and can involve life-threatening organs, such as alveolar hemorrhage. Rapidly progressive glomerulonephritis which manifests pathologically as crescentic glomerulonephritis, carries a high risk of progression to end stage renal disease (ESRD) or dialysis-dependent risk (1). GPA is a form of antineutrophil cytoplasmic antibody (ANCA)-associated vasculitis and usually manifests itself as either cytoplasmic ANCA-positive (C-ANCA) or anti-protease 3 (anti-PR3) antibody-positive (2, 3). Histopathologically, infiltration of lymphocytes, plasma cells and multinucleated giant cells and necrotizing granulomatous inflammation of small and medium-sized blood vessels are mostly manifested in cells and tissues.

Compared with normal people, naive B cells increased during AVV activity and GPA remission period (4, 5), and naive B cells were more sensitive to BCR stimulation during GPA activity period (6). The decrease in memory B cells observed in some disease activities may be due to increased differentiation into plasma cells, which migrated to sites of inflammation instead of peripheral blood (5). Besides, it is reported a decrease in percentage of B1 like B cells during the active phase of vasculitis (7) and a recovery in the number of B cell during remission (8). B cells also secreted pro-inflammatory cytokines such as IL-6 and TNF, which can reduce the increase in anti-inflammatory activity of T cells (9).B-lymphocyte stimulator (BLyS, also known as B-cell activating factor, BAFF) and proliferation-inducing ligand (APRIL) are members of the tumor necrosis factor (TNF) family, which are key factors in the survival and maturation of B-lymphocytes, and have also been implicated in the pathogenesis of a wide range of human autoimmune disorders (10). It has been shown that even in antineutrophil cytoplasmic antibody-associated vasculitis (AAV) remission, elevated expression of TACI in CD19+ cells, immature B cells, and the proportion of plasmablast (PB)/plasma cell (PC) is accompanied by persistently high levels of BAFF and APRIL in serum (11). Persistent abnormalities in BAFF/APRIL signaling may lead to disease recurrence (12). Telitacicept is a novel fully human TACI-Fc recombinant fusion protein targeting B lymphocyte stimulators. It can block BLyS to inhibit the development and maturation of aberrant B cells and block APRIL to inhibit antibody production by aberrant plasma cells (11, 13–16).

As we reported below, we observed favorable safety and efficacy of telitacicept in combination with conventional therapies (hormones, immunoglobulins, and CYC) for the treatment of patients with severe, rapidly progressive renal impairment who with pathological manifestations of crescentic nephritis with plasma cell infiltration.

## 2 Case presentation

**Figure 1:**
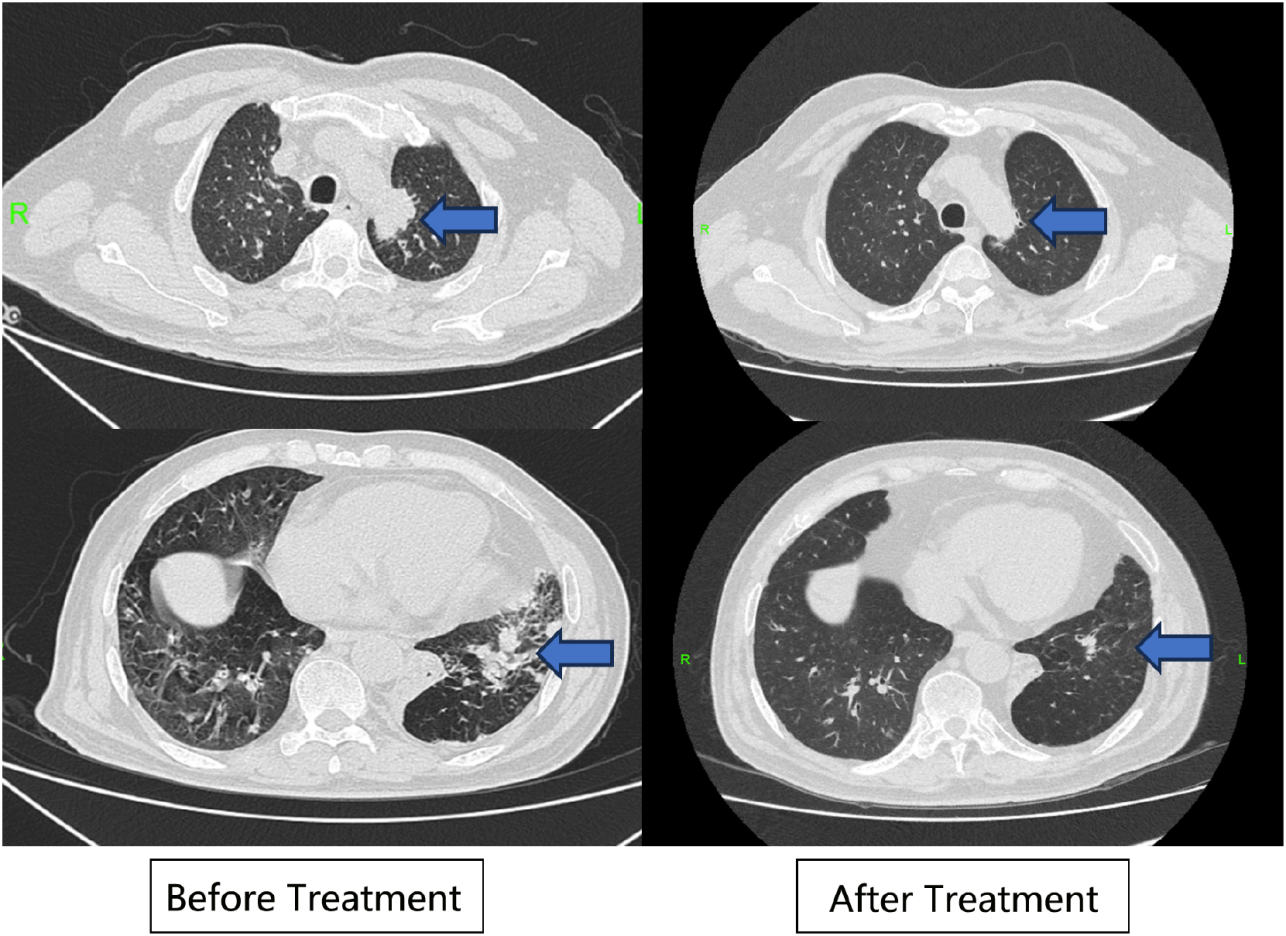
Lung CT. The blue arrows refer to the lesions in lung image before and after treatment.

**Figure 2:**
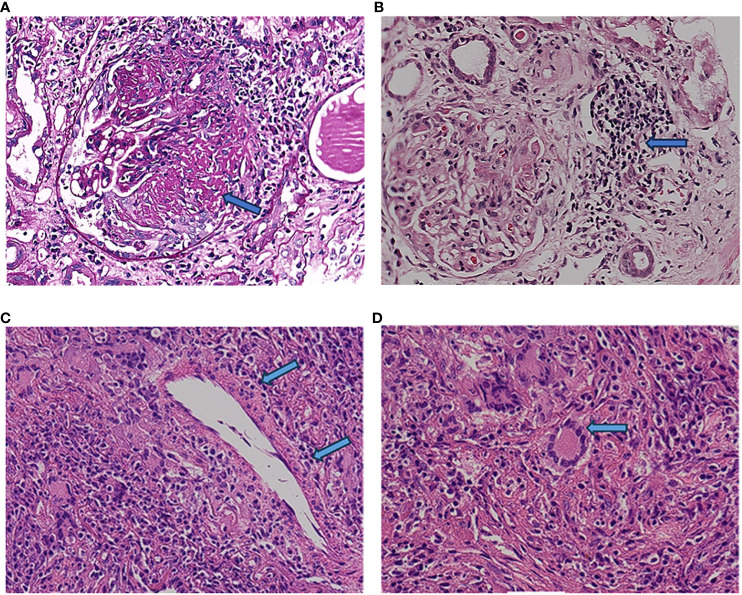
Renal histopathology. The labels in **(A, B)** refer to the large cell crescent body and plasma cell infiltration in kidney pathology, while the labels in **(C, D)** refer to plasma cell infiltration and typical multinuclear giant cells in lung pathology.

**Figure 3:**
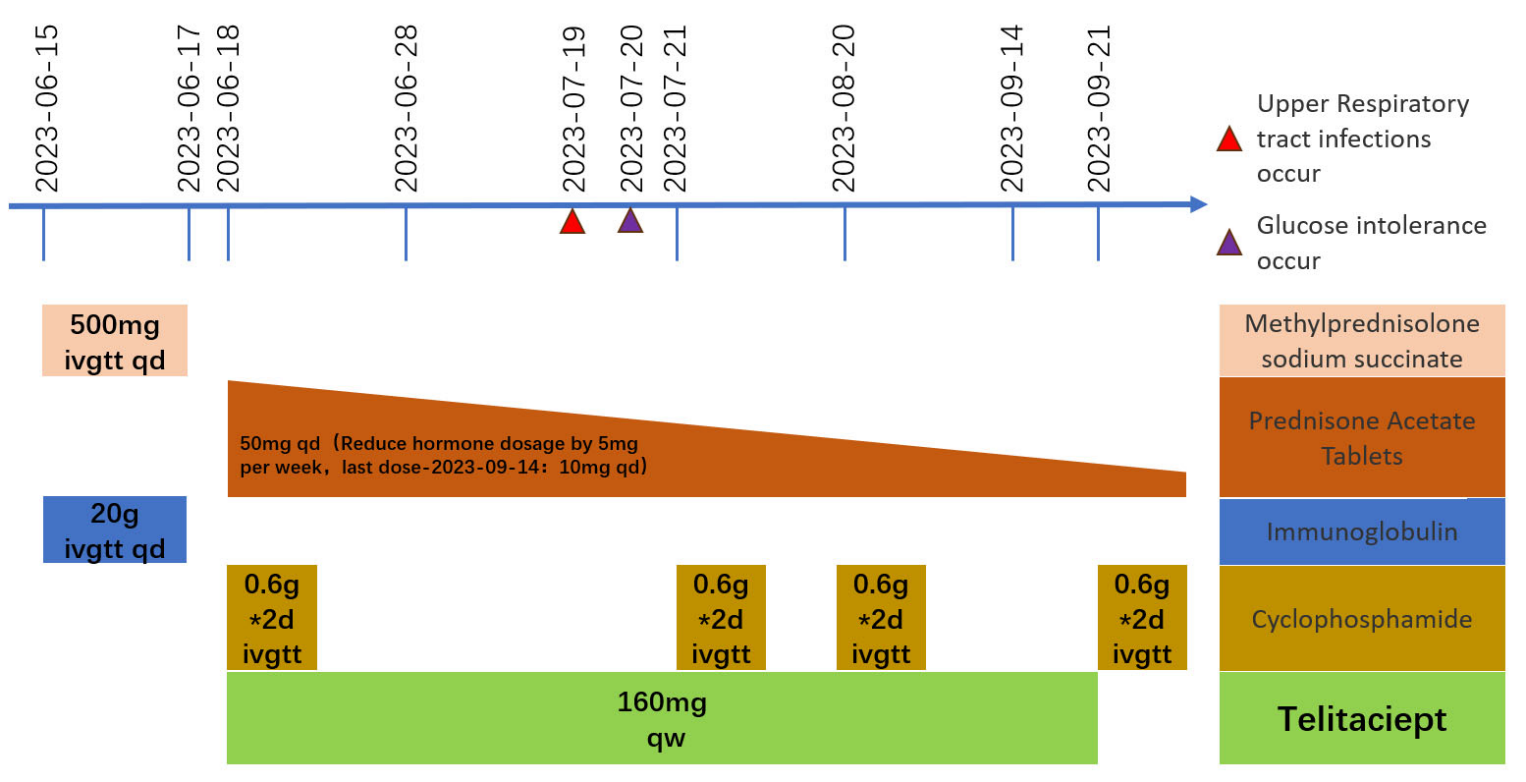
Medication flow chart.

A 64-year-old man was admitted to the Seventh Affiliated Hospital of Sun Yat-sen University in June 2023 because of hearing loss for 3 months, cough for 2 months and creatinine increase for 2 weeks. The patient had been in his usual state of health until 3 months before admission, when hearing loss, symmetry in both ears, and yellow discharge from the external ear canal developed. One month later, the patient presented cough, a small amount of white sputum, low fever, headache, and no hemoptysis. The creatinine of the patient was 119 umol/L during the visit in another hospital 15 days before admission. He had lost 10 kg of weight in the past 2 months and smoked 20-40 cigarettes a day for 30 years. Upon admission, he had normal vital signs, height 166cm, weight 79kg, BMI 28.6 kg/m^2^. He developed hearing loss and no purulent discharge in the external ear canal. No obvious abnormality was found in cardiopulmonary examination. Admission laboratory examination: Blood examination: white blood cells (WBC) 12.13 ×10^^9^/L (reference range 3.5-9.5*10^^9^/L), hemoglobin (Hb) 90 g/L (reference range 130-175 g/L); Urine examination: 5-10 red blood cells/HP were detected by microscopy (reference range 0-2/HP); 24-hour urinary protein quantification: 0.5-0.7 g/24h (reference range 0-150 mg/24h); Erythrocyte sedimentation rate (ESR): 29 mm/h (reference range 0-43.5 mm/h) C-reactive protein 118 mg/L (reference range<10 mg/L); Biochemical indicators: Urea 16.3 mmol/L (reference range 3.1-8.0 mmol/L), Creatinine (CRE) 382 μmol/L (reference range 57-97 umol/L), albumin 27 g/L (reference range 40-55 g/L). pANCA (+/-) (reference range -), MPO (++) (reference range -); Immunoglobulin indicators: IgG 19.9 g/L (reference range 7.0-16.0 g/L), IgA 1.35 g/L (reference range 0.7-4.0 g/L), IgM 0.436 g/L (reference range 0.4-2.3 g/L). Imaging examination: Lung CT: 1. Multiple nodules and spots in both lungs, and neoplastic lesions cannot be excluded; 2. Multiple lymph nodes enlargement in the mediastinum (see **Figure 1**). Sinus CT: Paranasal sinusitis; Bilateral middle ear mastoiditis. Pathological examination: Renal perforation pathology on June 16, 2023: Immunofluorescence IgM (+/-), CD38 (+), the rest were all negative. Light microscopy considered ANCA-associated vasculitis renal damage (with plasma cell infiltration), and crescent nephritis (crescent body: 11/13, macrocellular crescent body 6/11) were considered (see **Figures 2A, B**). Pathology of left inferior lobe mass on June 14, 2023: chronic granulomatous inflammation was considered, GPA cannot be excluded with abundant plasma cells can be seen in the tissue (see **Figures 2C, D**). The diagnosis was GPA with progressive glomerulonephritis. The BVAS score was 29.Shock therapy with methylprednisolone sodium succinate (500 mg, qd, ivgtt) combined with immunoproteins (20 g, qd, ivgtt) was given. After 3 days, the treatment was changed to prednisone (0.6 mg/kg/d, equivalent to prednisone (50 mg, qd)), which was reduced by 5 mg per week. A CYC regimen (0.6 g ivgtt) was also given, with a cumulative 1.2 g per month regimen. A telitacicept regimen (160 mg, qw) was also added (see **Figure 3**).

### 2.1 Outcome and follow-up

**Figure 4:**
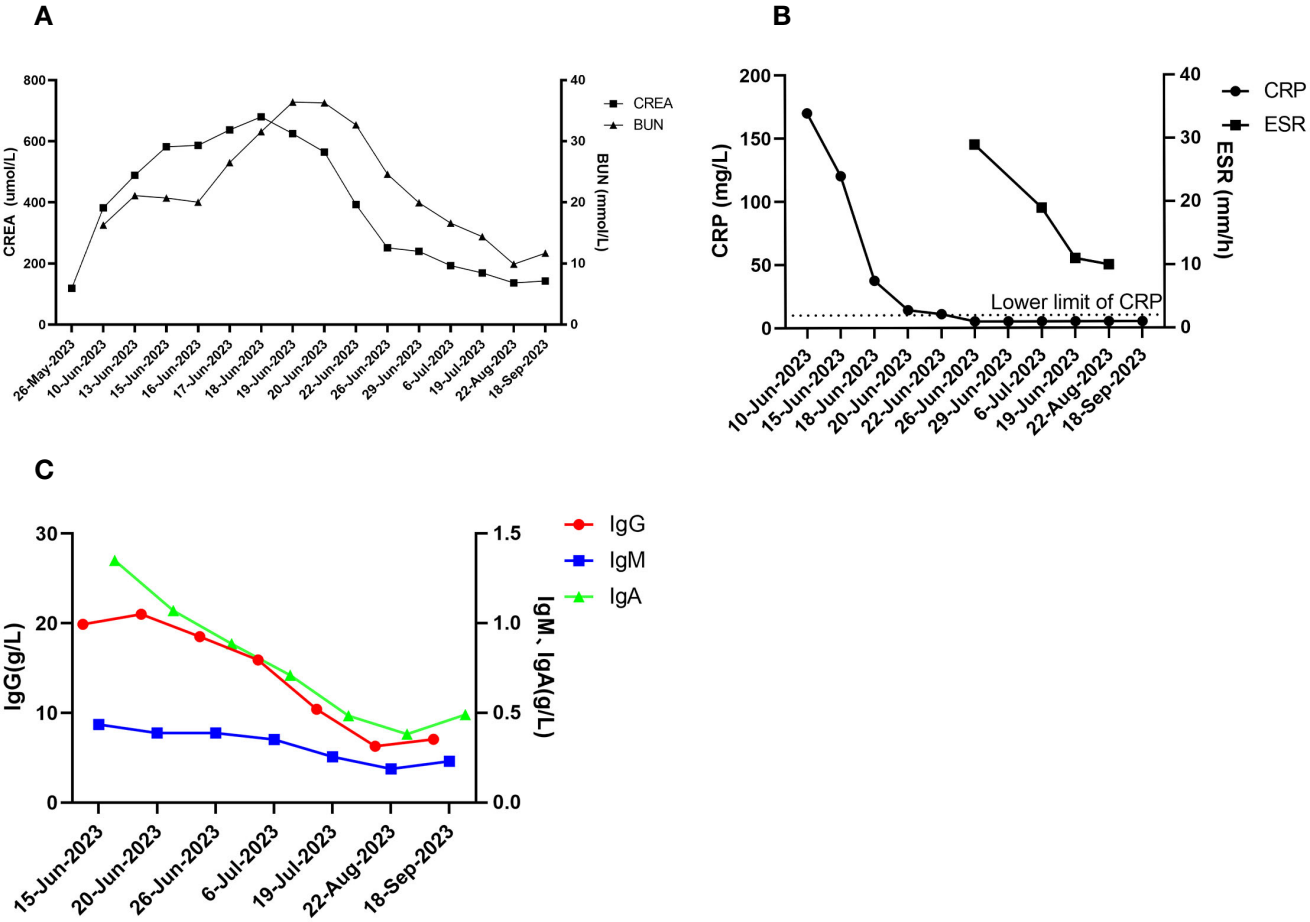
Post-treatment effects. **(A)** creatinine and urea nitrogen; **(B)** CRP and ESR; **(C)** immunologic markers (IgG, IgA, IgM).

The patient was followed up in July 2023, August 2023, and September 2023, respectively. The patient’s hearing gradually returned to normal and creatinine gradually decreased to 130-140 umol/L (reference range 57-97 umol/L) (see **Figure 4A**). The levels of CRP and ESR decreased (see **Figure 4B**), while the levels of IgG, IgA and IgM gradually decreased (see **Figure 4C**). The double pneumonia lesions were absorbed than before (see **Figure 1**). The patient developed acute upper respiratory tract infection at week 4 during follow-up, improved after moxifloxacin (0.4 g, qd, po*5d) anti-infection, and developed abnormal glucose tolerance at week 4. By September 2023, the treatment was updated to prednisone 10mg qd, total CYC 4.8g, and telitacicept 160mg qw ih.

## 3 Discussion

Systemic vasculitis diseases are considered as diseases with multisystem involvement. GPA commonly involves the ear, nose, upper and lower respiratory tracts (1). In 2022 EULAR updated guidelines for ANCA-associated vasculitis, life- or organ-threatening clinical manifestations are defined as glomerulonephritis, pulmonary hemorrhage, meningeal damage, central system damage, retro-orbital disease, cardiac damage, mesenteric involvement, and mononeuritis multiplex (3).

It has been shown that GPA is predominantly characterized by C-ANCA antibody positivity (73.3-76%) and PR-3 ANCA (74-82.2%) antibody positivity, and also by P-ANCA antibody positivity (9.8-13%) and MPO-ANCA antibody positivity (5-8.1%) (2, 3). In severe GPA involving multiple systems, the proportion of C-ANCA and PR-3 ANCA was higher with 78% and 76%, respectively (17). In the present case report, the patient exhibited rare positive antibodies to P-ANCA and MPO-ANCA. Moreover, it has been shown that GPA patients with MPO-ANCA antibody positivity have more severe glomerulosclerosis, interstitial fibrosis, tubular necrosis, tubular atrophy, and epithelial cell infiltration than GPA patients with PR-3 ANCA-positive (18).

In the 2022 EULAR update of the guidelines for ANCA-associated vasculitis, glucocorticoids in combination with RTX or CYC regimens are recommended for patients with organ- or life-threatening GPA (3). According to 2021 KDIGO guidelines, a combination of rituximab and cyclophosphamide may also be considered in patients with significantly reduced or rapidly declining glomerular filtration rate (GFR) (Serum creatinine [SCr] > 4 mg/dl [>354 μmol/l]) (19). Several studies have described the use of various combinations of RTX and CYC. The European Vasculitis Association (EUVAS) study has found that this combination regimen reduced the risk of progression to ESRD, recurrence, and death (20). A combination of RTX and CYC was also used to limit the use of glucocorticoids. Studies of RTX in combination with CYC for ANCA-associated vasculitis in patients with severe kidney disease reported a remission rate of up to 84% at 5 months with a relatively rapid glucocorticoid taping program and a similar rate of serious infection to RAVE therapy (21, 22). In addition, combination therapy has been used successfully in prospective cohorts. Limiting glucocorticoid use to 2 weeks or less resulted in excellent remission rates (>90%) even in patients with severe kidney disease (23).

RTX is a monoclonal antibody that targets CD20, which is an antigen expressed on the surface of B cells (24).The renal pathology and lung pathology of the patient in this case report showed plasma cell infiltration, and it was considered that RTX only targets peripheral blood circulating B cells, not plasma cells, and long-lived plasma cells are still the source of ANCA-producing antibodies (21, 25). Telitacicept inhibits the development and maturation of abnormal B cells by blocking BLyS, and also inhibits antibody production by abnormal plasma cells by blocking APRIL, which in turn inhibits plasma cell survival (13–15, 26). Telitacicept is currently used for a variety of immune system disorders, including systemic lupus erythematosus, desiccation syndrome, and IgG4-associated diseases, as well as many other immune system disorders (17, 27–29)and various renal diseases (17, 30)). Based on the infiltrated plasma cells in kidney tissue and pulmonary nodule puncture in this patient, it was considered that RTX had no effect on the tissue infiltrated plasma cells, and telitacicept inhibited the further development and maturation of immature B cells by blocking BLyS. At the same time, by blocking APRIL, telitacicept can inhibit the differentiation of mature B cells into plasma cells, and affect the secretion of autoantibodies by autoreactive plasma cells, hence better controlling disease activities (31).In this patient, eGFR was <10 ml/min before treatment, and there was only one upper respiratory tract infection and postprandial glucose elevation during the treatment, without other serious complications. It is suggested that the combination of telitacicept with hormones and CTX is safe for the treatment of patients with severely decreased eGFR, and it can lead to a rapid reduction in hormone dosage and minimize hormone side effects.

GPA can present as multiple nodules in the lungs and enlarged hilar lymph nodes, and can be easily misdiagnosed as a lung tumor. In this case report, the patient was previously diagnosed with lung cancer with multiple metastases at an outside hospital. However, ANCA-associated vasculitis has a higher chance of being combined with a tumor compared to the normal population, and a case of peripheral T-cell lymphoma mimicking GPA has also been reported (32). Therefore, we still cannot slacken the tracking of tumors in such patients during follow-up.

Based on our review of the available literature, this is the first case reported in the literature in which telitacicept was applied in combination with CTX and hormone induction for the treatment of severe GPA. We believe that the improvement and remission of symptoms following treatment with this regimen can be partly attributed to its targeting of APRIL and BLyS, its multiphasic inhibition of B-cell and plasma cell survival and proliferation, and its reduction in the production of pathogenic antibodies *in vivo*. Our case report suggests that telitacicept in combination with CTX and hormones may be an effective and safe treatment for severe GPA. However, more clinical studies are needed to confirm the therapeutic effect of telitacicept in severe GPA.

## Data availability statement

The datasets presented in this article are not readily available because this is a case report. Requests to should be directed to Liqi Huang (huangliqi@sysush.com).

## Ethics statement

The studies involving humans were approved by Ethics Committee of Seventh Affiliated Hospital of Sun Yat-sen University. The studies were conducted in accordance with the local legislation and institutional requirements. The participants provided their written informed consent to participate in this study. Written informed consent was obtained from the individual(s) for the publication of any potentially identifiable images or data included in this article.

## Author contributions

LH: Investigation, Project administration, Writing – original draft. WL: Investigation, Visualization, Writing – original draft. YLiu: Investigation, Methodology, Writing – original draft. JZ: Resources, Writing – review & editing. YLi: Conceptualization, Resources, Writing – review & editing. ZZ: Conceptualization, Funding acquisition, Writing – review & editing. CT: Conceptualization, Funding acquisition, Writing – review & editing.
